# Psychogenic and neurogenic components in patients with psychogenic or neuropathic pruritus: PRURINEURO: A non‐interventional single‐centre prospective assay

**DOI:** 10.1002/ski2.267

**Published:** 2023-07-19

**Authors:** Marie Orliaguet, Emilie Brenaut, Anne‐Sophie Ficheux, Sylvie Boisramé, Laurent Misery

**Affiliations:** ^1^ LIEN University Brest Brest France; ^2^ Departments of Oral Surgery Brest University Hospital Brest France; ^3^ Departments of Dermatology Brest University Hospital Brest France

## Abstract

**Background:**

The causes of pruritus are multiple and commonly classified into six different categories: dermatological, systemic, neuropathic, psychogenic, mixed and idiopathic. In clinical practice, psychogenic and neurogenic mechanisms tend to be separated in the etiological diagnosis of neuropathic or psychogenic disorders; nevertheless, studies investigating the respective psychogenic and neurogenic components are lacking.

**Objective:**

The main objective of this work was to highlight the differences and potential common characteristics between psychogenic pruritus and neuropathic pruritus.

**Methods:**

This study was a noninterventional single‐centre prospective assay. Patients with neuropathic (NP) or psychogenic (PP) pruritus were proposed to participate. The psychogenic and neurogenic components of pruritus in these patients were evaluated using six validated questionnaires or criteria, namely, the diagnosis criteria of psychogenic pruritus, the NP5 questionnaire, the Brest Pruritus Qualitative Assessment Questionnaire, Hospital Anxiety and Depression Scale, Toronto Alexithymia Scale, and DN4i.

**Results:**

Twenty‐five patients with NP and 15 with PP were included. A difference between the two groups was observed for NP5, with mean scores of 2.8 ± 0.9 and 1.4 ± 1 for the NP and PP groups, respectively (*p* < 0.0001). For depression, the average score was 3.5 ± 3.9 for the NP group and 7.5 ± 5.1 for the PP group (*p* < 0.02).

**Conclusion:**

While neuropathic and psychogenic disorders are different diagnoses, neuropathic and psychogenic components may exist simultaneously in patients with NP or PP.

## Graphical abstract

Neuropathic and psychogenic are considered as separated categories of itch. This study shows that neuropathic and psychogenic components may exist simultaneously in patients with neuropathic pruritus or psychogenic pruritus.

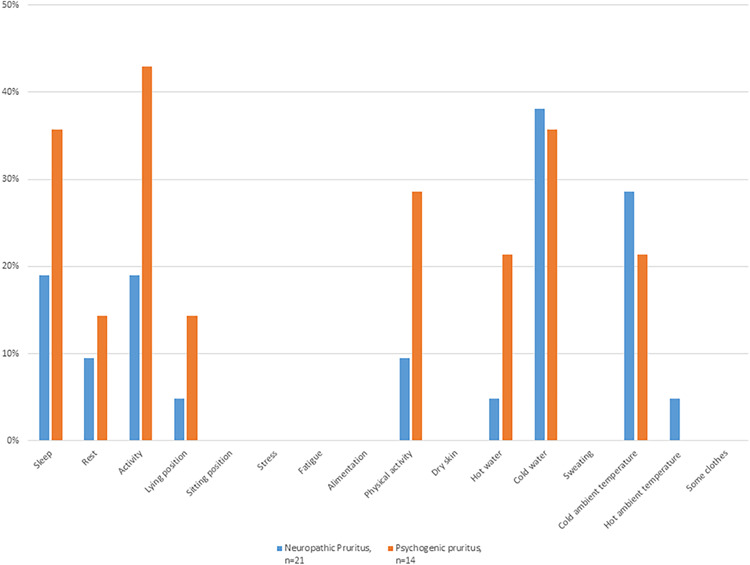

**What is already known about this topic?**

- Although there are separated definitions, it is sometimes difficult to make clinical distinction between neuropathic and psychogenic pruritus.

**What does this study add?**

- This paper shows that neuropathic and psychogenic components are frequently observed in patients with defined diagnoses. Consequently, a final diagnosis should be proposed cautiously.

## 1 INTRODUCTION

Pruritus, or itch, is defined as ‘an unpleasant sensation that causes an urge to scratch’, irrespective of whether scratching materialises.^1^, ^2^ Pruritus, which negatively affects psychological and physical aspects of life,^3^ is the most common symptom for consultation in dermatology.^4^, ^5^ Indeed, one‐third of the population reportedly experiences itch each week, and 10% of the population requires itch treatment.^6^ A large range of aetiologies of pruritus has been described and the International Forum for the Study of Itch (who considers the terms ‘itch’ and ‘pruritus’ synonymous) defines six categories: dermatological, systemic, neurological, psychogenic, mixed, and “other”.^7^ Hence, neurological or neuropathic pruritus is due to a neurological disorder while psychogenic pruritus is due to a psychological disorder.

Psychogenic pruritus (PP), or functional itch disorder, can be defined as ‘an itch disorder where itch is at the centre of the symptomatology and where psychological factors play an evident role in the triggering, intensity, aggravation, or persistence of the disease’.^8^ Because the diagnosis and definition of psychogenic pruritus lacks precision,^9^ the French Psychodermatology Group proposed 10 diagnostic criteria.^10^, ^11^, ^12^

Neuropathic itch refers to pruritus caused by neuronal or glial damage.^13^ Neuropathic pruritus can be localised (e.g., brachio‐radial pruritus or notalgia paraesthetica) or generalised (e.g., small fibre neuropathies).^13^ Neuropathic pruritus has several causes; it may arise from local nerve fibre compression or localised or generalised nerve fibre degeneration affecting different neuronal structures in the peripheral nervous system or central nervous system.^14^, ^15^

Scientific research on pruritus is in intensive development, and significant advances have been made in understanding its pathophysiology. In addition to the histaminergic pathway, a nonhistaminergic pathway has been described (proteinase‐activated receptors ‐ 2 ‘PAR‐2’‐dependent).^16^ Furthermore, Pruriceptors are selective itch receptors located in the skin.^17^

In daily practice, psychogenic and neurogenic aetiologies tend to be clearly separated in the diagnosis of pruritus. According to our previous work on burning mouth syndrome, we hypothesised that neuropathic and psychogenic factors could be frequently associated.^18^ The main objective of the present work, which was based on questionnaires, was to highlight the differences and the potential common characteristics between PP and NP to improve the differential diagnosis between these two pathologies. Secondary objectives were to describe the psychogenic and neurogenic characteristics of PP and NP to improve the understanding of the mechanism and, therefore, their management.

## 2 MATERIALS AND METHODS

### 2.1 Patients

This study was a non‐interventional single‐centre prospective assay. It was reviewed and approved by an instutitional review board, the Committee for the Protection of Individuals Sud‐Ouest and Outre‐Mer 1 (CPP 1‐21‐050 ID 12470). It was registered on ClinicalTrials.gov (NCT05024851).

The study population consisted of patients with PP or NP from the French Expert Centre on Pruritus in the dermatology department of the University Hospital of Brest in the previous 5 years. The diagnoses were assessed in usual conditions, from the medical history, clinical examination and para‐clinical elements. For the diagnosis of PP, the diagnostic criteria for functional itch disorder from the French Psychodermatology Group were used (three compulsory criteria: localised or generalised pruritus sine *materia*, chronic pruritus (>6 weeks) and the absence of a somatic cause; three additional criteria from the following seven items should also be present: a chronological relationship of pruritus with one or several life events that could have psychological repercussions, variations in intensity associated with stress, nocturnal variations, predominance during rest or inaction, associated psychological disorders, pruritus that could be improved by psychotropic drugs and pruritus that could be improved by psycho‐therapies.).^10^, ^11^, ^12^ For the diagnosis of NP, the diagnosis was made thanks to the association of pruritus with the occurrence of a neurological disorder (brachio‐radial pruritus, notalgia paraesthetica or small fibre neuropathies …) according to clinical manifestations and the result of imagery (local nerve fibre compression) or skin biopsy (nerve fibre degeneration).

All patients who met the following criteria were proposed to participate in the study: age 18 years and older; diagnosis of PP or NP; and ability to understand, agree to and sign the information and non‐opposition notices. The exclusion criteria were patients under legal protection (guardianship, curatorship) with acute or chronic conditions limiting the patient's ability to participate in the study.

All patients who consulted for PP or NP were reached by phone; the objectives of the study were explained, and they were asked if they still had a pruritus sensation. If the symptoms disappeared, the phone interview was then stopped. If a patient still experienced pruritus sensation, he or she was included in the study after his or her agreement. Two questionnaires were administered during the phone call. The non‐opposition notices and four other questionnaires were sent by postal mail to the patient. The questionnaires are described in the following section.

### 2.2 Questionnaires/outcomes

The psychogenic and neurogenic components of the included patients were evaluated using six validated questionnaires or criteria, namely, the diagnosis criteria of functional itch disorder or psychogenic pruritus, NP5 questionnaire, Brest Pruritus Qualitative Assessment Questionnaire, Hospital Anxiety and Depression Scale (HADS), Twenty‐Item Toronto Alexithymia Scale (TAS‐20) and Douleur Neuropathique 4 questionnaire (DN4i).

#### 2.2.1 Diagnostic criteria of functional itch disorder or psychogenic pruritus

To clarify the diagnosis of psychogenic pruritus, the French Psychodermatology Group proposed 10 diagnostic criteria (FPDG criteria).^10^, ^11^ It includes three compulsory criteria: localised or generalised pruritus sine materia, chronic pruritus (>6 weeks) and the absence of a somatic cause. Three additional criteria from the following seven items should also be present: a chronological relationship of pruritus with one or several life events that could have psychological repercussions, variations in intensity associated with stress, nocturnal variations, predominance during rest or inaction, associated psychological disorders, pruritus that could be improved by psychotropic drugs and pruritus that could be improved by psychotherapies.

#### 2.2.2 NP5 questionnaire

NP5 is a diagnostic tool dedicated to NP, where a score of two out of five criteria allows to discriminate NP pruritus from non‐NP, with a sensitivity of 76% and a specificity of 77%. The five questions (one point if positive response) are as follows: (1) is your pruritus associated with twinges?; (2) is your pruritus associated with burning?; (3) is your pruritus worse with activity?; (4) is your pruritus worse with stress; and (5) is your pruritus relieved by cold ambient temperature?^19^

#### 2.2.3 Brest Pruritus Qualitative Assessment Questionnaire

This questionnaire allows a qualitative assessment of pruritus, specifying its chronology, location, intensity (VAS10, 0: no itch sensation, 10: the worst itch sensation), characteristics and effect on daily activities of the itching.^20^

#### 2.2.4 Hospital anxiety and depression scale (HADS)

The HADS is a tool to detect anxiety and depressive disorders. It encompasses 14 items (seven referring to anxiety and seven to depressive dimension), scored from 0 to 3, thus yielding two scores between 0 and 21. A score less than or equal to 7 corresponds to an absence of anxiety or depressive disorders; a score between 8 and 10 indicates questionable symptomatology and a score greater than or equal to 11 confirms the existence of anxiety or depressive disorders.^21^, ^22^, ^23^

#### 2.2.5 Twenty‐item Toronto alexithymia scale (TAS‐20)

Alexithymia is a personality trait characterised by difficulties in understanding and describing one's own emotions. In the general population, the prevalence of alexithymia is approximately 10%.^24^ ^,^ ^25^, ^26^ The TAS‐20, a self‐administered psychometric questionnaire, is a validated tool to assess alexithymic traits with 20 items. Each item is rated on a five‐point Likert scale (from 1 = strongly disagree to 5 = strongly agree) corresponding to a total score ranging between 20 and 100. The following validated cutoff values were considered: ≤51 non alexithymia, 52 to 60 borderline (possible alexithymia) and ≥61 alexithymia.^27^, ^28^

#### 2.2.6 Douleur neuropathique 4 questionnaire (DN4i)

The DN4 is a questionnaire for the rapid assessment of patients with putative neuropathic pain. The short form, abbreviated DN4 (DN4i), contains two questions:

- ‘Does the pain have one or more of the following characteristics?’, including burning, painful cold, and electric shocks.
- ‘Is the pain associated with one or more of the following symptoms in the same area?’ including tingling, pins and needles, numbness, and itching.

For each positive item, one point is assigned, making the possible score range from 0 to 7. A DN4i score equal to or greater than 3 suggests the nature of neuropathic pain syndrome with a sensitivity of 78% and a specificity of 81%.^29^

### 2.3 Data analysis

The different quantitative variables were expressed as the mean and standard deviation (±SD), and the qualitative variables were expressed as an absolute value and percentage. Fisher's exact test was used to compare data for qualitative variables, and Student's *t* test was used for quantitative variables. For each test, a *p* value < 0.05 was considered statistically significant.

## 3 RESULTS

### 3.1 Demographic and pruritus characteristics (Table 1)

Forty patients were included in the study: 25 in the NP group and 15 in the PP group. Three patients did not return the questionnaire (NP: *n* = 2; PP: *n* = 1). Some patients did not answer all questions; they were consequently excluded from the analysis of the corresponding questionnaire(s) (refer to the numbers indicated in each table).

**TABLE 1:** Demographic and pruritus diagnosis characteristics of the included patients.

	Neuropathic pruritus (*n* = 25)	Psychogenic pruritus (*n* = 15)	*p value*
Age (years) mean ± SD	63 ± 12.1	62.2 ± 16	0.86
Gender (female/male) *n* (%)	15/10 (60%/40%)	5/10 (33.3%/66.7%)	0.19
Pruritus *n* (%)			
Generalized ‐ SFN	18 (72%)	/	
Localized ‐ BRP	5 (20%)	/	
Localized –Notalgia paresthetica	2 (8%)	/	
Psychogenic	/	15 (100%)	
NP5 score			
Mean ± SD	2.8 ± 0.9	1.4 ± 1	0.0001*
Score ≥ 2, *n* (%)	24 (96%)	7 (46.7%)	0.0006*
Diagnosis criteria of functional itch disorder			
Three compulsory criteria *n* (%)	0	15 (100%)	
Additional criteria mean ± SD (min‐max)	2.8 ± 1.5 (1–7)	5.9 ± 1 (4–7)	
≥ 3 additional criteria *n* (%)	13 (52%)	15 (100%)	0.001*
Current treatments for pruritus *n* (%)			
Emollient	7 (28%)	4 (26.7%)	
Topical corticosteroid	1 (4%)	5 (33.3%)	
Antihistamine	7 (28%)	1 (6.7%)	
Pregabalin	3 (12%)	0	
Gabapentin	3 (12%)	0	
Antidepressant	3 (12%)	7 (46.7%)	
Benzodiazepine	1 (4%)	6 (40%)	
Sleeping pill	1 (4%)	1 (6.7%)	

Abbreviations: BRP, Brachioradial pruritus; min‐max:, minimum‐maximum; SD, standard deviation; SFN, small fibre neuropathy.

*p < 0.05.

A difference between groups was observed for NP5, with mean scores of 2.8 ± 0.9 and 1.4 ± 1 for the NP and PP groups, respectively (*p* < 0.0001). For the NP group, 96% of the patients had a score ≥2 (PP: 46.7%; *p* = 0.0006).

Concerning the criteria of functional itch disorder, all patients in the PP group had at least three additional criteria, with an average of 5.9 ± 1 (NP: 52%; *p* < 0.001), while 52% of the NP group had at least three additional criteria.

### 3.2 Brest Pruritus Qualitative Assessment Questionnaire (Table 2)

The majority of the patients had pruritus for more than 1 year (NP: 95.2%; PP: 85.8%). For the majority of the patients, the symptomatology onset was progressive (NP: 57.1%; PP: 71.4%), and the rhythm was intermittent (NP: 66.7%; PP: 64.3%), without any statistically significant difference between groups. The maximum intensity of itching sensation was 7.9 ± 2 for the NP group and 7.8 ± 1.8 for the PP group (*p* = 0.91), and the minimum was 1.7 ± 2.4 for the NP group and 1.9 ± 2.4 for the PP group (*p* = 0.82). The mean intensity was 5.2 ± 2.2 for the NP group and 5.2 ± 1.7 for the PP group (*p* = 0.97).

**TABLE 2:** Characteristics of pruritus in neuropathic and psychogenic pruritus.

	Neuropathic pruritus (*n* = 21)^a^	Psychogenic pruritus (*n* = 14)^a^	*p value*
Duration, *n* (%)			
Weeks	0	1 (7.1%)	
Months	1 (4.8%)	1 (7.1%)	
Years	20 (95.2%)	12 (85.8%)	
Symptomatology onset, *n* (%)			0.49
Brutal	9 (42.9%)	4 (28.6%)	
Progressive	12 (57.1%)	10 (71.4%)	
Rhythm, *n* (%)			1
Continuous	7 (33.3%)	5 (35.7%)	
Intermittent	14 (66.7%)	9 (64.3%)	
Frequency of pruritus sensation, *n* (%)			
Everyday	13 (61.9%)	11 (78.6%)	
Almost daily	5 (23.8%)	3 (21.4%)	
Weekly	1 (4.8%)	0	
Monthly	1 (4.8%)	0	
Rarely	1 (4.8%)	0	
Intensity mean ± SD (min‐max)			
In the worst moment	7.9 ± 2 (3–10)	7.8 ± 1.8 (4–10)	0.91
In the best times	1.7 ± 2.4 (0–10)	1.9 ± 2.4 (0–7)	0.82
On average	5.2 ± 2.2 (0–10)	5.2 ± 1.7 (2–8)	0.97

Number of itch localisations per patient	Neuropathic pruritus (*n* = 23)^a^	Psychogenic pruritus (*n* = 14)^a^	*p value*
Mean ± SD	6.4 ± 4.4	6.9 ± 3.9	0.70
Range (min‐max)	1–16	2–14	

Abbreviations: min‐max, minimum‐maximum; SD, standard deviation.

^a^ Not all participants answered all questions.

**FIGURE 1:**
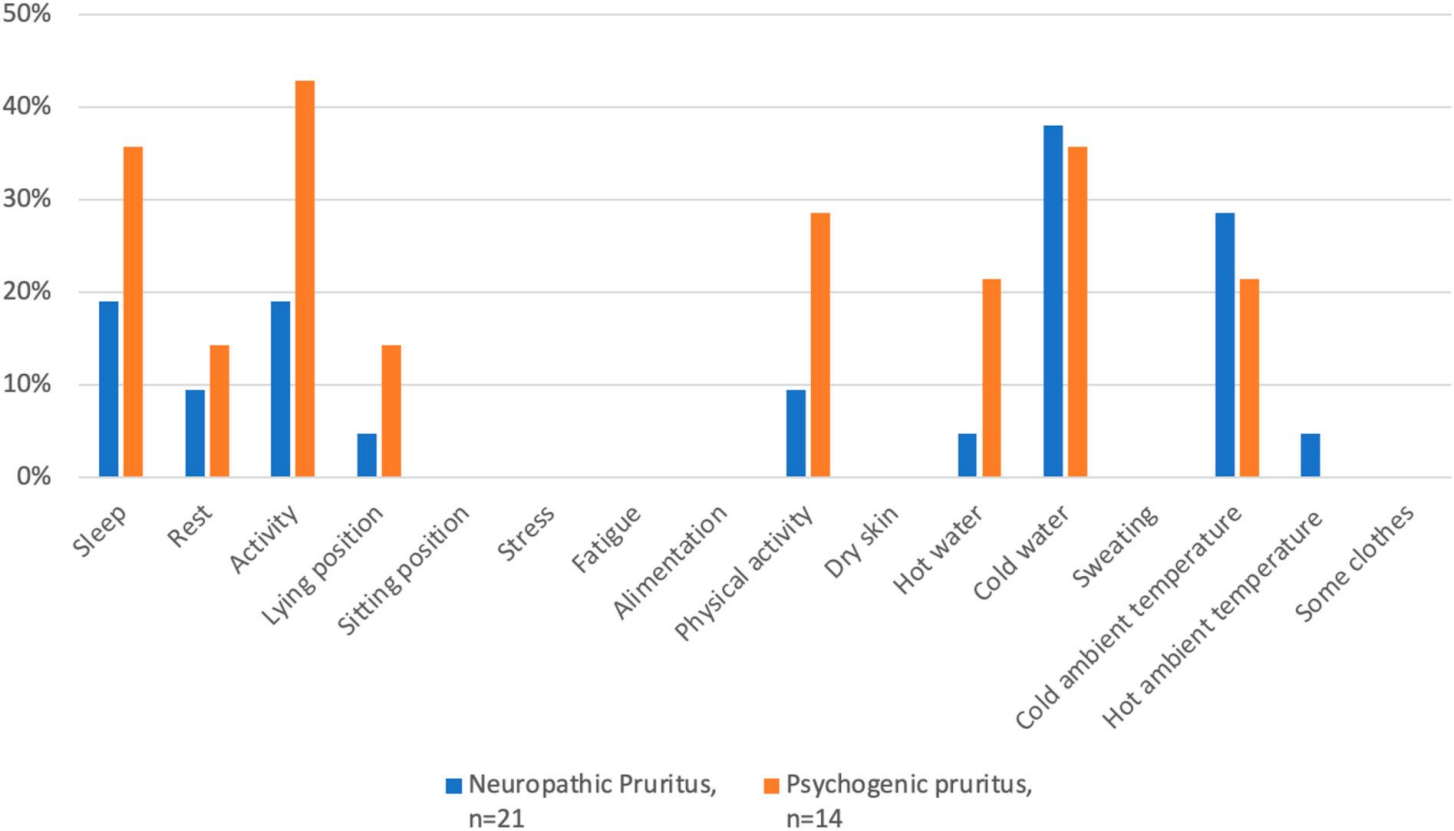
Pruritus alleviating factors in neuropathic and psychogenic pruritus. Not all participants answered all questions.

**FIGURE 2:**
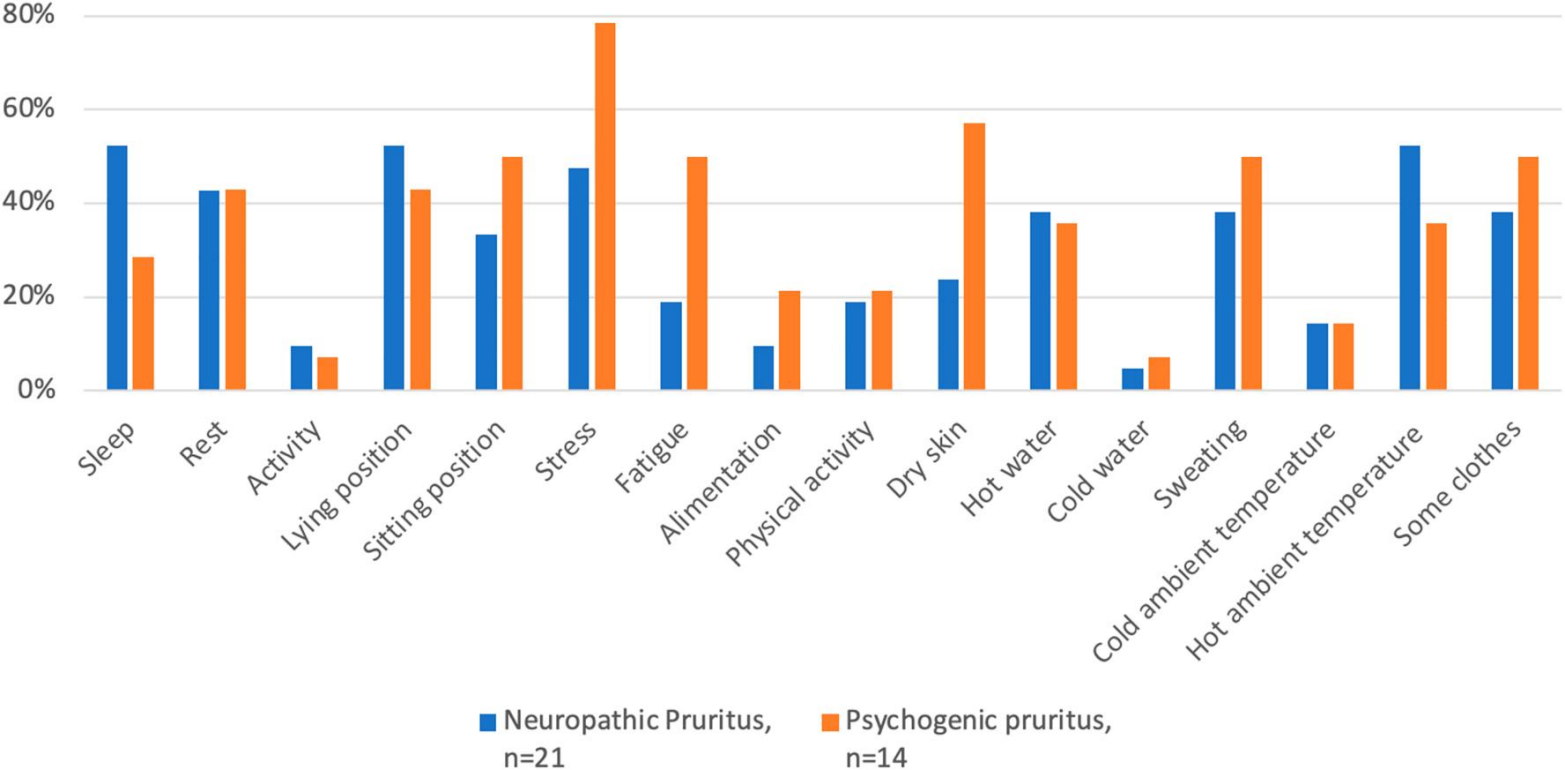
Pruritus worsening factors in neuropathic and psychogenic pruritus. Not all participants answered all questions.

Regarding scratching, 85.6% of the NP patients and 85.8% of the PP patients scratched themselves often or very often (Suppl. Figure S1). In the NP group, 35% experienced scratching as very unpleasant, while 55% found it moderately or very pleasant. In the PP group, very unpleasant and very pleasant feeling sensations were expressed by 33.3% of the respondents (Suppl. Figure S2). For the majority of the PP respondents, a predominance of itching sensations at the end of the day was observed. Regarding the NP group, most of the patients did not have an itching sensation during the morning; half of them often felt an itching sensation in the evening (Suppl. Figure S3). Depending on the patient, the itching localization was variable; nevertheless, a statistically difference between groups was observed for the back (NP: 60.9%; PP: 92.9%; *p* = 0.05). For the NP group, 78.3% of patients had an upper arm localization, and no patients from the PP group felt an itching sensation on the plantar surface (Suppl. Figure S4). No significant difference was found in the characteristics accompanying the itching. Heat sensation in the itchy area was observed in 63.6% of the NP group. Tingling was the main characteristic found in the two groups (NP: 72.7%; PP 92.3%). No patients experienced a cold sensation in the itchy area (Suppl. Figure S5). Regarding factors that worsened or alleviated pruritus, stress (NP: 47.6%; PP: 78.6%; *p* = 0.08) and fatigue (NP: 19%; PP: 50%; *p* = 0.07) exacerbated symptoms in both groups. Cold water and cold ambient temperature relieved pruritus sensation in both groups. Activity and sleep alleviated pruritus sensation in 42.9% and 35.7% of patients in the PP group, respectively (Figures 1 and 2).

### 3.3 HADS, TAS‐20 and DN4i questionnaires (Table 3)

Regarding anxiety, the average HADS anxiety subscale score was 7.1 ± 4.1 in the NP group and 8.6 ± 5.2 in the PP group; three NP and five PP patients had an overall score ≥11 in favour of anxiety disorder. For depression, the average score was 3.5 ± 3.9 for the NP group and 7.5 ± 5.1 for the PP group (*p* = 0.02); three patients in the PP group had an overall score ≥11 in favour of depression disorder.

**TABLE 3:** Hospital anxiety and depression scale, TAS 20 and DN4i scores.

HADS	Neuropathic pruritus (*n* = 21)^a^	Psychogenic pruritus (*n* = 14)^a^	*p value*
Anxiety subscale score			
Mean ± SD	7.1 ± 4.1	8.6 ± 5.2	0.36
Range (min‐max)	0–16	2–17	
Score, *n* (%)			
score ≤ 7	13 (61.9%)	7 (50%)	
8 ≤ score ≤10	5 (23.8%)	2 (14.3%)	
score ≥ 11	3 (14.3%)	5 (35.7%)	0.22
Depression subscale score			
Mean ± SD	3.5 ± 3.9	7.5 ± 5.1	0.02*
Range	0–16	2–18	
Score, *n* (%)			
score ≤ 7	17 (80.9%)	8 (57.2%)	
8 ≤ score ≤10	3 (14.3%)	3 (21.4%)	
score ≥ 11	1 (4.8%)	3 (21.4%)	0.28

	Neuropathic Pruritus (*n* = 19)^a^	Psychogenic pruritus (*n* = 12)^a^	*p value*
TAS20			
Mean ± SD	63.3 ± 13.7	59.5 ± 10.3	0.39
Range (min‐max)	38–96	42–75	
Score, *n* (%)			
≤ 51 non alexithymia	4 (21.1%)	3 (25%)	
52 to 60 borderline	5 (26.3%)	3 (25%)	
≥ 61 alexithymia	10 (52.6%)	6 (50%)	1

	Neuropathic Pruritus (*n* = 23)^a^	Psychogenic pruritus (*n* = 14)^a^	*p value*
DN4i			
Mean ± SD	3.3 ± 1.9	3.3 ± 1.6	0.92
Range (min‐max)	0–7	0–7	
Score, *n* (%) score < 3	8 (34.8%)	2 (14.3%)	
Score ≥ 3	15 (65.2%)	12 (85.7%)	0.26

Abbreviations: min‐max, minimum‐maximum; SD, standard deviation.

^a^ Not all participants answered all questions.

*p < 0.05.

According to the TAS‐20, 52.6% of the NP patients and 50% of the PP had alexithymic traits; the two groups had a similar mean score (NP: 63.3 ± 13.7; PP: 59.5 ± 10.3; *p* = 0.39).

Of the 37 patients, 15 patients (65.2%) in the NP group and 12 (85.7%) in the PP group had a DN4i score in favour of neuropathic pain (score ≥3). The DN4i scores ranged from 0 to 7 in both groups, with mean scores of 3.3 ± 1.9 and 3.3 ± 1.6 for the NP and PP groups, respectively (*p* = 0.92).

## 4 DISCUSSION

The cohort consisted of 15 PP and 25 NP patients. The psychogenic and neurogenic components of these patients were evaluated using diagnostic criteria of functional itch disorder or psychogenic pruritus and the NP5 questionnaire and four validated questionnaires sent by postal mail to the patient, namely, the Brest Pruritus Qualitative Assessment Questionnaire, HADS, TAS‐20, and DN4i. The qualitative characteristics of NP and PP were very similar, justifying the use of NP5 and the FPDG to try to distinguish them.

Notably, DN4i, which is a questionnaire to identify neuropathic pain, is not a powerful tool in this context since DN4i score was more frequently higher or equal than 3 in patients with PP than those with NP.

For the NP5, 96% of the NP patients had a score ≥2; this result confirms that NP5 is a relevant tool for the diagnosis of NP, although question 4 (on stress) might induce confusion with PP because this item is globally common to these two types of pruritus. A score in favour of neuropathic pain was found in 65.2% of the NP group and 85.7% of the PP group, with similar scores in both groups, which shows that DN4i is appropriate for the diagnosis of neuropathic pain but not NP because pain is not present in all patients. Concerning the criteria of functional itch disorder, all PP patients had at least three additional criteria, while 52% of the NP group had at least three additional criteria too, suggesting that an additional psychogenic component exists in NP or that NP has psychological consequences. The high score of anxiety in both groups confirms this hypothesis.

According to the TAS‐20, 52.6% of the NP group and 50% of the PP group had alexithymic traits, with similar mean scores. Hence, alexithymia does not seem to be involved in PP psychopathology. Thanks to HADS scores, we identified higher scores of anxiety in patients with PP. For depression, the average score was dramatically lower in the NP group than the PP group, suggesting that depression is mainly involved in the pathophysiology of PP.

The present work presents several limitations. Although it is monocentric study on 40 patients, this study provides some clues for further surveys at larger levels. This first study gave the opportunity to highlight problems in the protocol that should be considered in further studies. Hence, the choice of using paper questionnaires sent by postal mail led to a lack of data due to the incomplete filling in of certain items.

## 5 CONCLUSION

Based on the results from this study, neuropathic and psychogenic components may exist simultaneously in patients with NP or PP. This overlap is probably related to the role of the nervous system in both disorders. However, the NP5 and FPDG criteria are relevant tools for discriminating NP and PP. Depression appears to be highly associated with PP.

## AUTHOR CONTRIBUTIONS

Marie Orliaguet performed the study and wrote the paper, Laurent Misery designed the study, Laurent Misery and Sylvie Boisramé supervised the study, Emilie Brenaut and Anne‐Sophie Ficheux analysed results, all authors reviewed and approved the paper.

## CONFLICT OF INTEREST STATEMENT

None declared.

## ETHICS STATEMENT

This study was reviewed and approved by an institutional review board, the Committee for the Protection of Individuals Sud‐Ouest and Outre‐Mer 1 (CPP 1–21‐050 ID 12470). It was registered on ClinicalTrials.gov (NCT05024851).

## Supporting information

Supporting Information S1

## Data Availability

The data that support the findings of this study are available from the corresponding author upon reasonable request.
