# Supplementary material for: Psychogenic and neurogenic components in patients with psychogenic or neuropathic pruritus: PRURINEURO: A non‐interventional single‐centre prospective assay
Source: Skin Health Dis. 2023 Jul 19;3(6):e267. doi: 10.1002/ski2.267 (PMC10690707; doi:10.1002/ski2.267)
Supplement: Supplementary file 1 — Supporting Information S1 [file SKI2-3-e267-s001.docx]

Supplementary figure 1. Scratching frequency

Not all participants answered all questions.

Supplementary figure 2. Pleasurability of scratching

*Not all participants answered all questions.*

Supplementary figure 3. Moment of the day of itching sensation

*Not all participants answered all questions.*

Supplementary figure 4. Localization of itching

*Not all participants answered all questions.*

Supplementary figure 5. Characteristics accompanying the itching

*Not all participants answered all questions.*
